# Calix[3]arene-Analogous Metacyclophanes: Synthesis, Structures and Properties with Infinite Potential

**DOI:** 10.3390/molecules25184202

**Published:** 2020-09-14

**Authors:** Md. Monarul Islam, Paris E. Georghiou, Shofiur Rahman, Takehiko Yamato

**Affiliations:** 1 Chemical Research Division, Bangladesh Council of Scientific and Industrial Research (BCSIR), Dhanmondi, Dhaka 1205, Bangladesh; mmipavel@yahoo.com; 2 Department of Chemistry, Memorial University of Newfoundland, St. John’s, NL A1B 3X7, Canada; 3 Aramco Laboratory for Applied Sensing Research, King Abdullah Institute for Nanotechnology, King Saud University, Riyadh 11451, Saudi Arabia; msrahman@mun.ca; 4 Department of Applied Chemistry, Faculty of Science and Engineering, Saga University, Honjo-Machi 1, Saga 840-8502, Japan; yamatot@cc.saga-u.ac.jp

**Keywords:** calix[3]arenes, metacyclophanes, calixarene-analogous metacyclophanes, inherent chirality, host-guest chemistry

## Abstract

Calixarene-analogous metacyclophanes (CAMs) are a special class of cyclophanes that are cyclic polyaromatic hydrocarbons containing three or more aromatic rings linked by one or more methylene bridging groups. They can be considered to be analogues of calixarenes, since, in both types of molecules, the component aromatic rings are linked by methylene groups, which are meta to each other. Since the prototype or classical calix[4]arene consists of four benzene rings each linked by methylene bridges, which are also meta to each other, it can be considered to be an example of a functionalized [1.1.1.1]metacyclophane. A metacyclophane (MCP) that consists of three individual hydroxyl-group functionalized aromatic rings linked by methylene groups, e.g., a trihydroxy[1.1.1]MCP may therefore, by analogy, be termed in the broadest sense as a “calix[3]arene” or a “calix[3]arene-analogous metacyclophane”. Most of the CAMs reported have been synthesized by fragment coupling approaches. The design, synthesis and development of functionalized CAMs, MCPs, calixarenes and calixarene analogues has been an area of great activity in the past few decades, due their potential applications as molecular receptors, sensors and ligands for metal binding, and for theoretical studies, etc. In this review article, we focus mainly on the synthesis, structure and conformational properties of [1.1.1]CAMs, i.e., “calix[3]arenes” and their analogues, which contain three functionalized aromatic rings and which provide new scaffolds for further explorations in supramolecular and sensor chemistry.

## 1. Introduction

**Figure 1:**
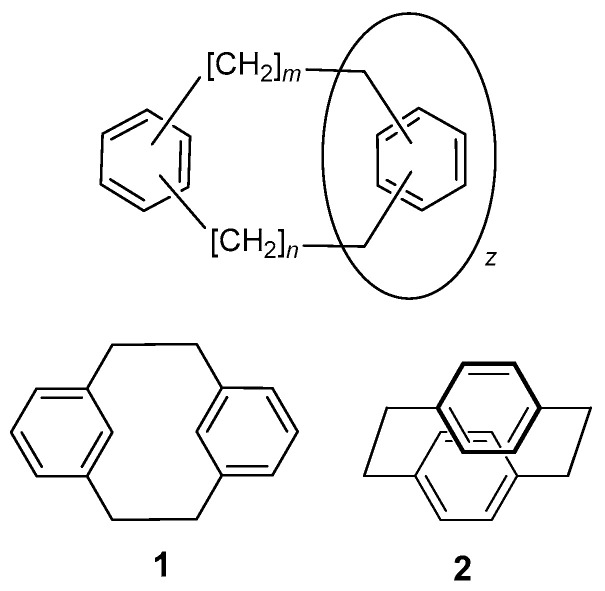
Top*:* Generic cyclophane structure with the *ortho*-, *meta*- or *para*-substitution of the phenyl rings and where *m*, *n* and *z* are variable. Compound **1** is [2.2]metacyclophane and **2** is [2.2]paracyclophane, where *m* = *n* = 2 and *z* = 1.

Cyclophanes are an important class of bridged aromatic hydrocarbons, consisting of one or more aromatic units linked by methylene group chains in such a way that the aromatic units form part of a macrocycle. A generic synthetic structure of cyclophanes in which two or more aromatic groups are linked via their *ortho, meta* or *para* positions by variably sized methylene groups is shown in Figure 1. These molecules have been extensively pursued and studied for a variety of different considerations [1,2]. Cyclophane chemistry can be considered to have been initiated by Pellegrin [3] who, in 1899, reported the synthesis of [2.2]metacyclophane (i.e., [2,2]MCP) **1** by Wurtz coupling of 1,3-bis(bromomethyl)benzene. The real development of the chemistry of cyclophanes, however, is well-acknowledged to have occurred after Cram and Steinberg’s synthesis of [2.2]paracyclophane **2** in 1951 [4].

**Figure 2:**
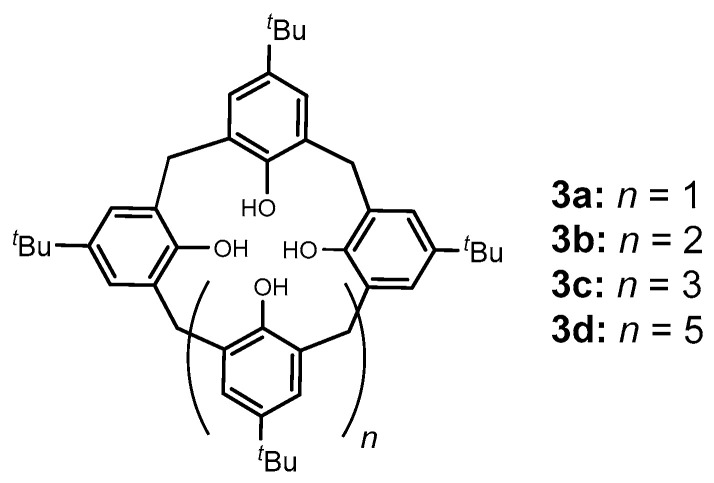
Basic structural motifs of calix[*n*]arenes **3a**–**d**.

**Figure 3:**
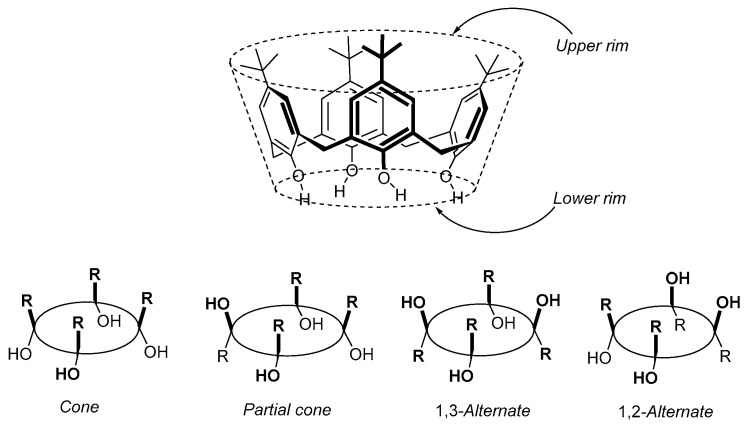
General structure of p-substituted calix[4]arene (e.g., R = *t-*Bu) and schematic representations of its formal conformational isomers.

On the other hand, calixarenes such as **3a**–**d** (Figure 2), which can be considered to be examples of [1*_n_*]metacyclophanes (where *n* = 4, 5, 6 or 8, respectively), have also become an important class of compounds since Zincke and Zeigler’s first report in 1944 of a cyclic tetrameric compound (later confirmed to be **3a**) from the base-induced reaction of *para*-substituted *tert*-butylphenol with formaldehyde [5]. The importance of this class of compounds derives from the fact that calixarenes have “basket”- or “cup”-like 3-D topologies, with the hydroxyl groups forming a narrower or “lower” rim due to the strong intermolecular hydrogen bonds. The opposite rim is termed the “upper” rim and is wider due to the steric repulsion of the *para*-substituents and the orientation of the aromatic rings although for the larger calix[*n*]arenes (*n* > 6); however, their shapes or conformations can vary or be more complex. As a result, these molecules can be selectively functionalized at either rim and they can serve as building blocks for a variety of applications, such as, for example, ionic or molecular receptors. Gutsche and coworkers pioneered and developed methodologies for synthesizing *p*-*tert*-butylcalix[4]arene **3a** [6] (also commonly and inaccurately referred to simply as “calix[4]arene”, which should strictly be confined to the de-*tert*-butylated derivative of **3a**), *p*-*tert*-butylcalix[5]arene **3b** [7], *p*-*tert*-butylcalix[6]arene **3c** [8], and *p-tert*-butylcalix[8]arene **3d** [9] (recently, Haase reported a highly selective and high-yield synthesis for the production of calix[8]arenes [10]) as major products. Gutsche’s syntheses were achieved using *p-tert*-butylphenol and formaldehyde under different but reproducible reaction conditions, which have allowed these compounds, and derivatives thereof, to become easily accessible to be widely studied. Derivatives of **3a** are well-known to be able to exist in four different major conformationally immobile conformers known as *cone*, *partial-cone*, *1,3*-*alternate*, and *1,2*-*alternate* conformers (Figure 3) and which can be selectively generated and isolated by introducing different functional groups onto the lower rim of **3a** [11].

Many calixarene-based molecular receptors have been synthesized and studied, but calix[4]arenes and its derivatives with amide, ketone and ester functionalities at the lower rim, which have shown significant cation affinity [12,13], have received the most attention to date. In general, modifications of calixarenes have been via functionalization of their lower or upper rims. The spirodienone route [14] and Lhtoak’s mercuration [15] route, however, are notable exceptions for effecting the direct modifications to the basic calix[4]arene skeleton not specifically involving the functionalization of the upper and/or lower rims, or via convergent fragment-based approaches. Since there are many excellent reviews and monographs that have been published on calixarenes, and their more typical analogues, including homocalixarenes [16], homooxacalixarenes [17,18], azacalixarenes [19] and hexahomoazacalixarenes [20], and other types [21,22] the reader is referred to these so will not be further elaborated upon here. Instead, for this review, we have chosen to emphasize the MCP skeleton that has been extensively explored as a versatile and stable platform, or scaffold, for functionalization and study. In particular, we present a specific focus upon those functionalized MCPs, which in the broadest sense, can be considered to be directly analogous with calix[3]arenes in general. A review of the syntheses and properties of cyclophanes incorporating three aromatic units is presented below.

## 2. Calixarene-Analogous MCPs Containing Three Aromatic Rings: Calix[3]arene Analogues

**Figure 4:**
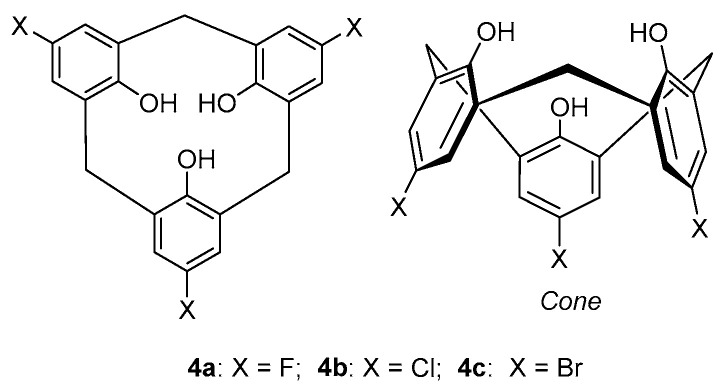
General structures of the [1.1.1]metacyclophane (MCP) calixarene analogues **4a**–**c**.

Many reports during the past few decades have dealt with the modification and studies of the properties of calix[*n*]arenes, particularly those in which *n* = 4,5,6 or 8, but not many have concerned calix[3]arenes containing only three aromatic rings, i.e., calix[3]arenes or [1.1.1]MCPs (or [1*_n_*]MCPs), with the exception of the hexahomoxacalix[3]arenes in which the bridging groups are one or more -CH_2_OCH_2_- groups [17,18]. [1.1.1]MCPs cyclophanes, which incorporate only three aromatic rings, have been a major focus of our group in recent years and provide useful molecular platforms, particularly for their synthesis and study of their conformational properties and molecular strain. In 1982, Moshfegh and co-workers reported the first synthesis of a series of functionalized [1.1.1]MCPs, i.e., *p*-halocalix[3]-arenes **4a**–**c** [23] (Figure 4).

These compounds which contained intra-annular hydroxy groups and halogens on the *para* positions of one, two or all three of the phenyl groups and which adopted *cone* type conformations, were obtained in 69–90% yields by the cyclocondensation(s) of the corresponding precursor mono- or dihalo-2,2’-dihydroxydiphenyl-methane with 2,6-bis(hydroxymethyl)phenol, or with 4-halo-2,6-bis(hydroxymethyl)phenols [23]. The antimicrobial properties of these compounds which were named as “phloroglucide” analogues were reported but surprisingly, to date, no other studies have been reported with these particular compounds.

## 3. Homocalixarene-Analogous MCPs Containing Three Aromatic Rings: Homocalix[3]arene Analogues

**Scheme 1:**
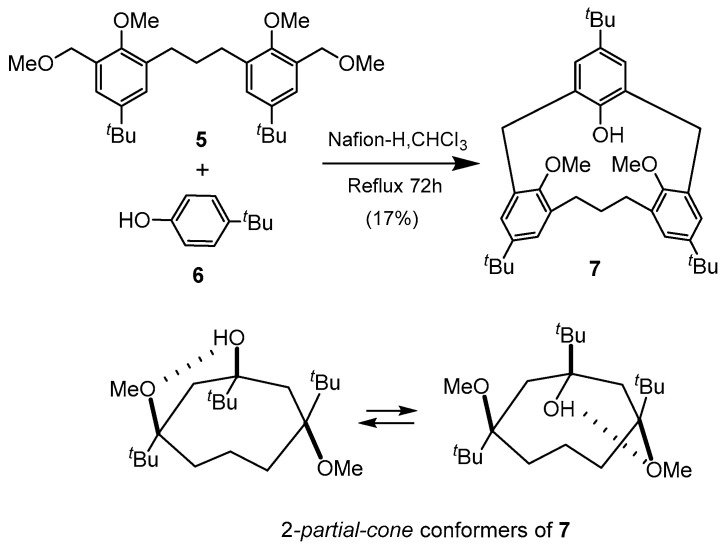
Synthesis of hydroxy[3.1.1]MCP calixarene analogue **7** and schematic representation of interconverting *2-partial-cone* conformer structures.

Expanded macrocyclic calixarene-analogous metacyclophanes (CAMs) containing polymethylene bridging groups such as [3.1.1]MCP 7 which contains a single trimethylene (−(CH_2_)_3_−) and two methylene bridges can be regarded as an example of an unsymmetrical homocalix[3]arene analogous MCP. Thus, 7 which can be considered to be an example of a [3.1.1]dihomocalix[3]arene-analogous MCP was synthesized using the Nafion-H catalyzed cyclobenzylation of **5** and *tert*-butylphenol **6** [24,25]. The trimethoxy and trihydroxy derivatives of **7** were also reported. The room temperature ^1^H-NMR spectrum of dimethoxy **7** revealed two sets of doublets for the methylene protons which implied that it is in an asymmetric “*2*-*partial-cone*” conformation, in which the two aromatic rings linked by the trimethylene group are anti to each other as shown in Scheme 1. However, at 80 °C in CDBr_3_, coalescence of the methylene protons indicates that there is conformational ring flipping above this temperature. A mechanism was proposed in which two possible structures results from the alternating hydrogen bonding formed between one of the methoxy groups and the hydroxyl group. The triol however was fixed in a typical *cone* conformation with all three hydroxyl groups syn to one another.

**Scheme 2:**
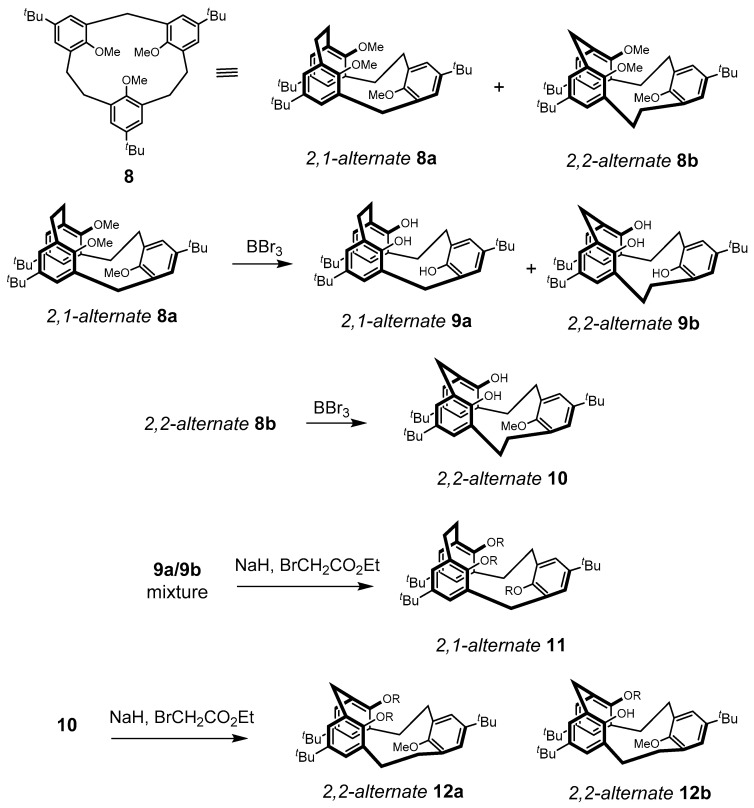
Conformers of [2.2.1]MCP dihomocalix[3]arene analogues **8**–**12** (R = CH_2_CO_2_Et).

Tashiro and coworkers reported the synthesis and conformational properties of the dihomocalix[3]arene containing one methylene and two dimethylene (−(CH_2_)_2_−) bridges, i.e., trimethoxy[2.2.1]MCP **8** and several of its derivatives. [26] The synthesis of **8**, which can also be considered to be a dihomocalix[3]arene analogue, was found to be a 2:1 mixture of asymmetric and symmetrical atropisoisomers **8a** and **8b**, respectively (Scheme 2). The synthesis was accomplished via the corresponding dithia[3.3.1]MCP precursor, which was transformed by the oxidation and sulfone pyrolysis-extrusion methodology under vacuum pyrolysis at 450 °C [27,28]. Based upon their ^1^H-NMR spectra, and with the use of Pirkle’s reagent, these molecules were shown to exist in alternate conformations designated as *2,1-alternate* **8a** and *2,2-alternate* **8b**, which could undergo rapid interconversion.

Trihydroxy[2.2.1]MCPs **9a** and **9b** were obtained by the simple demethylation of **8a**, but only dihydroxymethoxy[2.2.1]MCP **10** was obtained from the demethylation of **8b**, the remaining methoxy group presumably being sterically shielded from the BBr_3_ attack. Treatment of the mixture of **9a** and **9b** with NaH/ethyl bromoacetate afforded only MCP **11**. On the other hand, a 2:1 mixture of both MCPs **12a** and **12b** was obtained from similar treatment of *2,2-alternate* **10**. The single-crystal X-ray structures of both **8a** and **8b** further confirmed their structures. Tashiro’s group had previously reported the synthesis of trimethyl [2.2.2]MCPs [29]. These molecules, however, contained only intra-annular methyl groups and thus cannot be considered to be calix[3]arene-analogous MCPs, which, of course, typically contain hydroxyl or *O*-alkylated functional groups in the corresponding intra-annular positions.

**Scheme 3:**
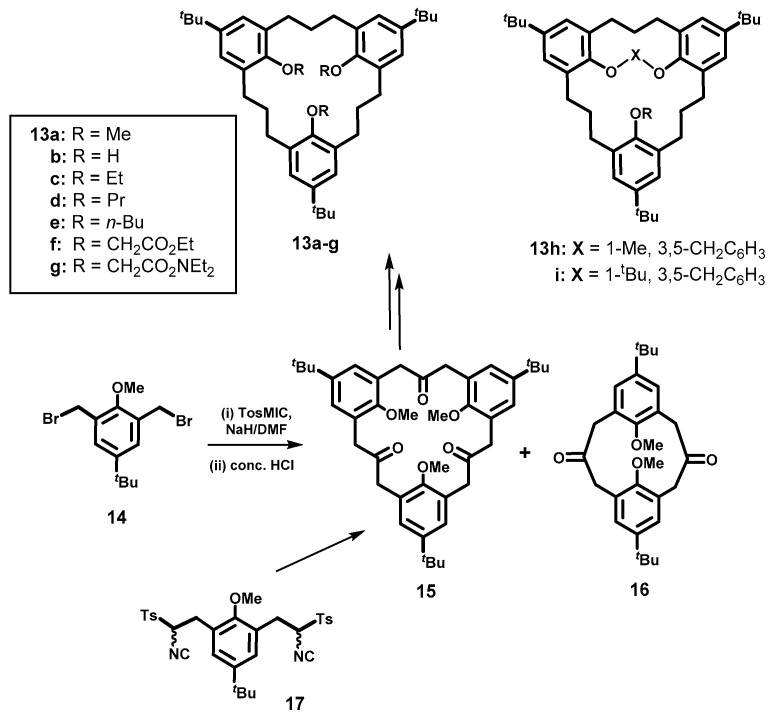
Hexahomocalix[3]arene analogue MCPs **13a**–**g** and capped **13h**–**i**.

The efficient syntheses of larger macrocyclic MCPs in which the aromatic groups are linked by varying larger numbers of −CH_2_− groups can also be effectively accomplished using two other non-sulfur extrusion processes, namely the low-valent zinc McMurry coupling reaction, or the TosMIC (*p*-tolylsulfonylmethyl isocyanide) [30,31] methodology that was used to great advantage by Vogtle’s group, for the synthesis of [3*_n_*]MCPs [32]. The synthesis, conformations and properties of several types of the larger homocalix[3]arene-analogue MCPs [33,34,35,36,37,38], using TosMIC has been reported by our group. For example, the synthesis of the symmetrical all-trimethylene-bridged hexahomocalix[3]arene analogue MCP, namely tri-*tert*-butyl-trimethoxy[3.3.3]MCP **13a**, was achieved via the TosMIC-NaH mediated cyclotrimerization reaction of 2,6-bis(bromomethyl)-4-*tert*-butylanisole **14**, followed by acid hydrolysis, to form trione **15** along with dione **16** in 22% and 10% yields, respectively (Scheme 3) [33], although better yields of **15** (68%) could be obtained using the 1 + 1 coupling reaction of bis-TosMIC adduct **17** with **14**, instead, as shown in Scheme 3. Wolff–Kishner reduction of **15** produced the desired tri-*tert*-butyl-trimethoxy[3.3.3]MCP **13a** in 86% yield. The demethylation of **13a** with BBr_3_ in dichloromethane gave the tri-*tert*-butyl-trihydroxy[3.3.3]MCP **13b** in 89% yield. The *O*-Alkylation of **13b** could be achieved in high yields with several alkyl halides (RX: R = Et, Pr, and *n*-Bu), in the presence of Cs_2_CO_3_ under reflux conditions in acetone, to predominantly yield the *partial-cone* conformers of derivatives **13c**–**g**. The *partial-cone* conformers of **13c**–**e** were formed exclusively, but, in the cases of **13f** and **13g**, the *partial-cone*:*cone* ratio fell from 95:5 to 67:33. On the other hand, when NaH was used instead as the base to form **13f** and **13g** also in high yields (>95%), the *cone* conformers were now exclusively formed. The authors rationalized their results by a mechanism in which ring-inversion of one of the rings could occur at different rates, leading to the formation of the *partial-cone* isomers with the ethyl, propyl and butyl derivatives. However, with the corresponding larger ethoxycarbonylmethoxy and *N,N*-diethylaminocarbonylmethoxy derivatives, a “metal-templating” effect with Cs^2+^ ion and more strongly with the Na^+^ ion, prevents complete ring inversion. Similar metal-templating effects had been noted previously in the case of *O*-alkylation reactions with calix[4]arenes. “Doubly-bridged” derivatives of **13b** [34] in which two of the hydroxy groups were capped using 3,5-bis(bromomethyl)toluene, or 1-*tert*-butyl-3,5-bis(bromomethyl)-benzene, Cs_2_CO_3_ and acetone under reflux conditions in acetone, was deduced to also be in a *partial-cone* conformation.

**Figure 5:**
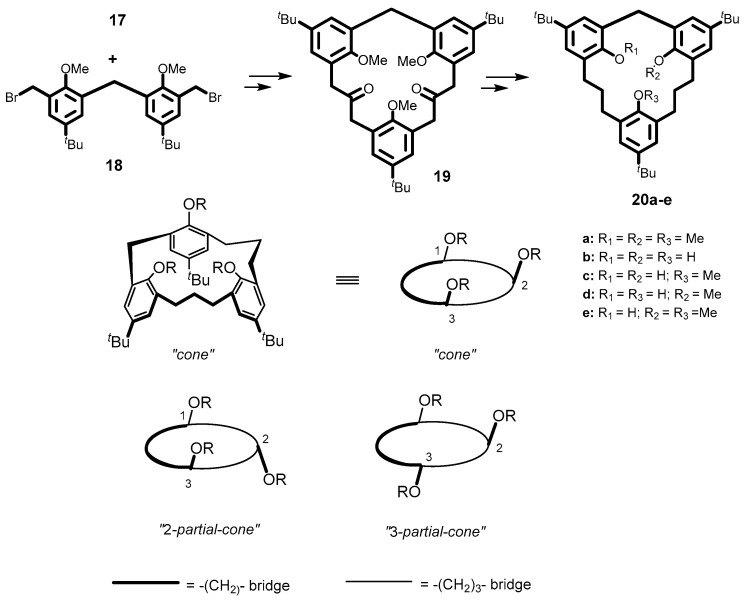
Synthesis and conformers of tetrahomocalix[3]arene analogue MCPs **20a**–**e**.

In a similar manner as with the synthesis of **13a**, the tetrahomocalix[3]arene-analogue MCP, tri-*tert*-butyl-trimethoxy-[3.3.1]MCP **20a** was synthesized by a NaH/DMF-mediated cyclocondensation of **17** and **18** followed by Wolff–Kishner reduction of the diketone intermediate **19** (Figure 5). The inherent chirality of the resulting *C*_1_-symmetrical **20e** was confirmed by its room temperature ^1^H-NMR spectrum with added Pirkle’s chiral shift reagent, which caused a splitting of the OMe groups and AB patterns corresponding to the methylene protons indicating a *2-partial-cone* conformation.

The demethylation of **20a** using tetramethylsilyl iodide (TMSI) in acetonitrile produced the corresponding trihydroxy[3.3.1]MCP, 20b, as expected, but when BBr_3_ in dichloromethane was used, the partial demethylation of **20a** resulted, forming only the dihydroxy[3.3.1]MCP, **20c**. Upon further treatment with TMSI/MeCN, however, **20c** afforded the trihydroxy[3.3.1]MCP **20b** [38]. Its ^1^H-NMR spectrum (in CDCl_3_) exhibits the signals for the hydroxyl groups at ~ δ 3.33 and 6.25 ppm, which is evidence for the formation of intramolecular hydrogen bonding and a *cone* conformation [38]. In contrast, both the ^1^H-NMR spectrum and a single crystal analysis confirmed the *2-partial-cone* conformation of macrocycle **20c**. Its ^1^H-NMR spectrum shows two sets of doublets at δ 3.61 and 4.36 ppm (*J* = 13.3 Hz) for the Ar*CH_2_*Ar methylene protons and a single peak at δ 1.72 ppm for the methoxy protons consistent with a *2-partial-cone* conformation. To date, no further studies on either the computational or supramolecular properties of these compounds have been reported.

## 4. Calixbenzofuran-Analogous MCPs: Calix[3]benzofuran Analogues

**Figure 6:**
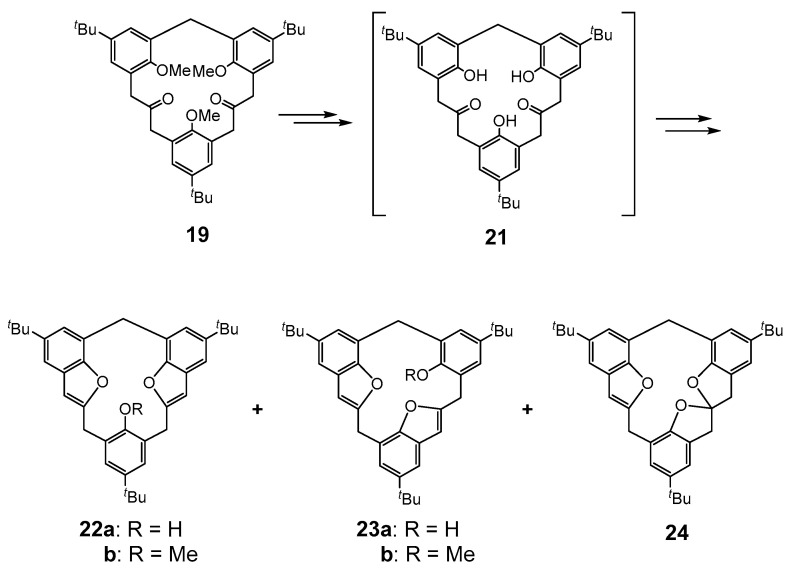
Calix[3]*di*benzofuran analogue MCPs **22**–**24.**

The demethylation of [3.3.1]MCP-dione **19** in acetonitrile with in-situ-generated TMSI produced a mixture of benzofuran ring-containing products, the symmetrical **22a** and unsymmetrical **23a** and a new spirobisdihydro-furan **24** in **24**, **45** and 5% yields, respectively (Figure 6) [39]. It is presumed that the trihydroxy-diketo intermediate **21** is first formed, and then undergoes intramolecular TMSI-mediated cyclizations to form the observed products [39,40,41]. Sawada and co-workers had previously reported that the treatment of tetramethoxy [2.1.2.1]MCPs with TMSI formed hemisphere-shaped calixarene analogues containing a dihydrobenzofuran ring [42,43]. The CDCl_3_^1^H-NMR spectrum of the symmetrical **22a** shows the hydroxyl signal at δ 6.54 ppm, indicating intramolecular hydrogen bonding between the hydroxyl and the oxygen of one of the benzofuran rings. The single-crystal X-ray structure of **22a** confirmed the intramolecular hydrogen bond as predicted from the ^1^H-NMR spectroscopic data, with a distance of 2.182 Å between the hydroxyl proton and one of the benzofuran oxygens. The unsymmetrical regioisomer **23a** is fixed in an asymmetrical hemispherically shaped *cone* conformation and the spirobisdihydrofuran **24** is presumed to have been formed by a nucleophilic attack on one of the formed benzofurans in **22a**.

**Figure 7:**
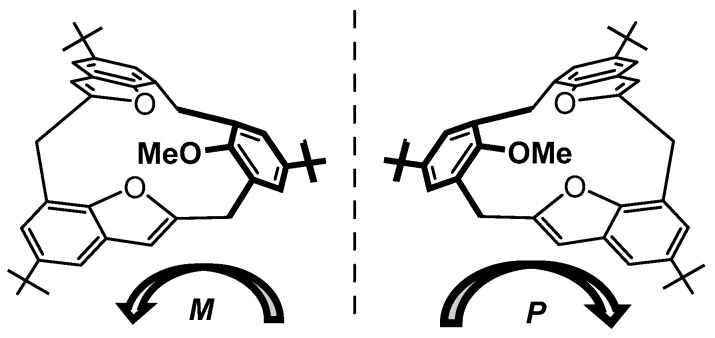
Schematic diagram of *M*-**23b** (**left**) and *P*-**23b** (**right**). Figure adapted from [39].

The *O*-methylation of **23a** with MeI/K_2_CO_3_ in acetone resulted in the folding of the arene ring into the macrocycle to form the non-symmetrical *partial-cone* methoxy[1.1.1]MCP **23b**, which is evident from the ^1^H signals observed for the bridge methylene hydrogen atoms. The methoxy group of **23b** is shifted to the high field as a singlet at δ 1.97 ppm due to its shielding by the benzofuran rings; these ^1^H signals correspond to an unsymmetrical *partial-cone* structure as depicted from density functional theory (DFT)-optimized energy structures. Its ^1^H-NMR spectra with added Pirkle’s reagent revealed the racemic mixture of *P*- and *M*-enantiomers (Figure 7). Single-crystal X-ray analysis of **23b** revealed that the macrocyclic skeleton adopts a highly asymmetric hemisphere-shaped *cone*-type conformation in which the methoxy group is pointed upwards and is exo to the two benzofuran rings, predicted from the ^1^H-NMR spectra. These molecules, therefore, by adopting curvature in their planar structures that have no symmetry planes in their three-dimensional representations, fit Szumna’s expanded definition of inherent chirality [44,45]. Others, including Böhmer [46,47] and Mandolini [48], had previously proposed and studied inherent chirality in calixarenes.

**Figure 8:**
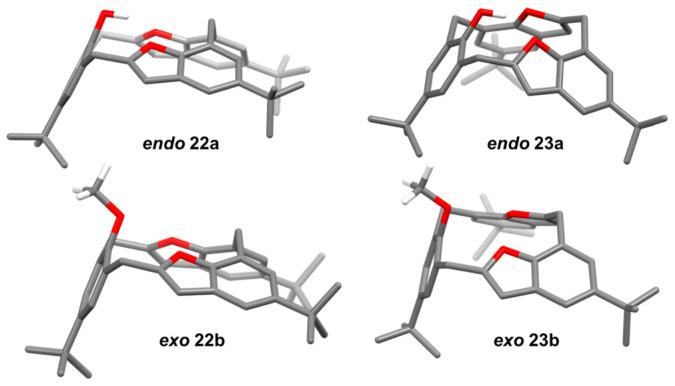
Geometry-optimized structures of *endo* **22a** and *endo* **23a**; *exo* **22b** and *exo* **23b**. All hydrogens except methoxy and phenolic hydrogens are omitted for clarity. Figure adapted and re-calculated from [39].

The DFT calculations conducted on these molecules using *Gaussian-09* [49] showed that the energy-minimized structures of the hemisphere-shaped *cone* conformers were in agreement with the observed single-crystal X-ray structures. The DFT gas phase calculations using B3LYP/6-31G(d) showed that of the regioisomers **22a** and **23a**, the latter of which was energetically more favored by 3.791 kJ mole^−1^. The DFT calculations of the corresponding conformations of methoxy derivatives **22b** and **23b** showed that the latter was also similarly more favored by 2.358 kJ mole^−1^, with the methoxy groups being favored to be in *exo* rather than *endo* orientations to the benzofuran rings, as shown in Figure 8.

**Figure 9:**
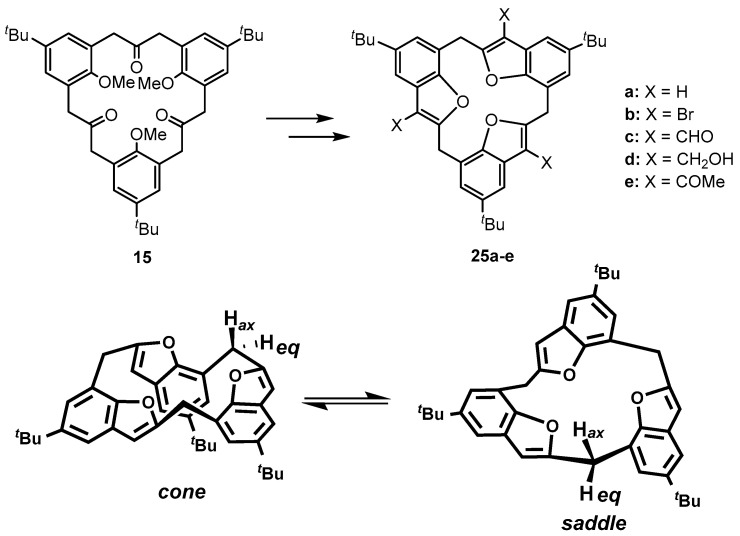
Calix[3]benzofuran analogue MCPs, **25a**–**e** and *cone* and *saddle* conformers of **25a** [40].

A series of derivatives **25b**–**e** of calix[3]benzofuran **25a** were synthesized using typical electrophilic aromatic substitution reactions to investigate the influence of the substituents on the conformations of the calix[3]benzofurans. The ^1^H-NMR spectra of **25a**–**e** reveal that they adopt diverse conformations in solution and in some cases undergo very fast conformational changes relative to the NMR time scale. For example, **25a** freely interconverts between *cone* and *saddle* conformers (Figure 9) in solution, but the tribromocalix[3]benzofuran **25b**, adopts a rigid *cone* type hemisphere-shaped symmetrical structure in the solid state. The triformyl derivative **25c** showed fast *cone*-*saddle* interconversion in solution, but, when the three formyl groups were reduced to form the corresponding trihydroxymethyl derivative **25d**, the molecule adopted a fixed-*cone* conformation as with **25b**. The acylation of **25a** to form **25e** once again led to the formation of a slowly-interconverting mixture of *cone-saddle* conformers (Table 1) [40].

**Figure 10:**
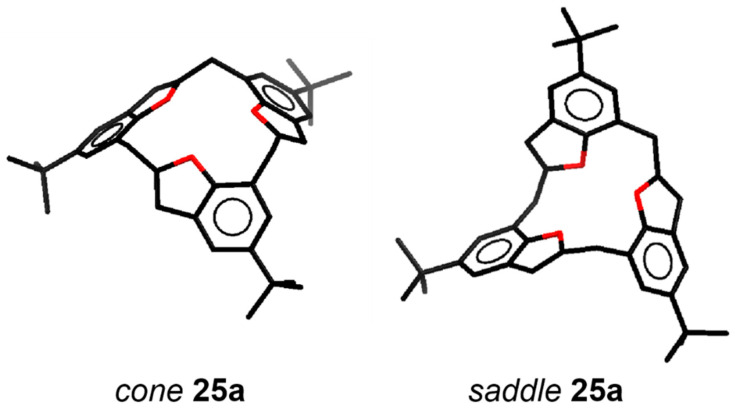
Geometry-optimized structures of the *cone* and *saddle* conformers of **25a**. Similar structures were obtained for the *cone* and *saddle* conformers of **25b**–**e** and are not shown.

Solvent and gas-phase DFT determinations with calix[3]benzofuran MCPs **25a**–**e** [40] reveal that both the *saddle* and corresponding *cone* conformers have lower ground-state energies in the solvent system than in the gas phase (Figure 10).

The calculations also show that, among the calix[3]benzofurans, the *saddle* conformers are energetically more favored than their corresponding *cone* conformers in both the solvent and gas-phase. The energy differences are in the following order: **23a** > **25e** > **25d** > **25c** > **25a** > **23b** > **22b**. Thus, by introducing the different groups at the furan moieties, their *saddle* conformers become energetically more favored, roughly according to the increasing size of the groups (i.e., COMe > CH_2_OH > CHO), except for **25b**. In the case of **25b**, there may be two factors influencing the stability of the *cone* conformers: bromine is electronegative in nature and also has greater electron density due to multiple lone-pairs of electrons. A single-crystal structure of **25b**, however, revealed it to be in a *cone* conformation in the solid state, but it should be noted that it co-crystallized with a well-defined chloroform molecule and with disordered solvent methanol molecules.

**Figure 11:**
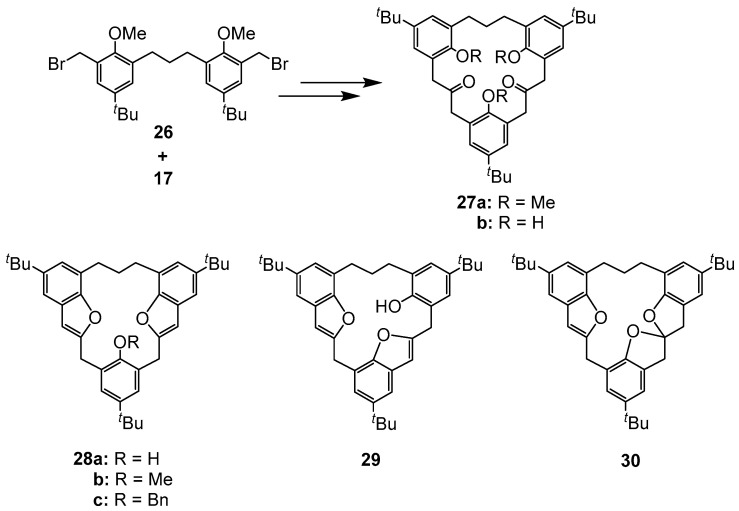
Structures of calix[3]benzofuran analogue MCPs **27**–**30**.

The cycloaddition reaction of **17** and **26**, followed by acidic work-up afforded [3.3.3]MCP **27a** (Figure 11) which, in turn, when treated with TMSI, generated in situ from chlorotrimethylsilane/sodium iodide in CH_3_CN, generated, instead of the expected trihydroxy **27b**, two calix[3]benzofuran[3.1.1]MCP-analogues **28a** and **29** in 52% and 7% yields, respectively [41]. The regioisomer of **28a**, namely the unsymmetrical **29**, however, could not be isolated, although it was detected in the ^1^H NMR spectra of the crude reaction mixture. The ^1^H-NMR spectrum of **28a** exhibits a single peak at δ = 4.09 ppm for the Ar*CH_2_*Ar methylene bridge protons and the trimethylene protons appeared as a broad multiplet at δ 2.01 and 2.98 ppm. The position of the hydroxyl group at δ 6.34 ppm is consistent with the existence of intramolecular hydrogen bonding between the hydroxyl group and the oxygen of one of the benzofuran rings. The formation of **30**, which contains the spirobisdihydrofuran moiety, is analogous to the spirobishydrofuran **24** described previously [41].

**Figure 12:**
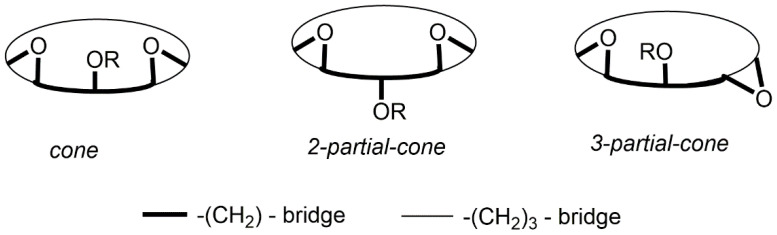
Schematic representations of the three basic possible conformers of **28** and **29**.

When **28a** was treated with MeI in the presence of K_2_CO_3_ in acetone methoxy[3.1.1]MCP **28b** was produced as its *cone*-conformer. This is evident from the ^1^H signals observed for the bridge methylene hydrogen atoms that are split and appear as a pair of doublets at δ = 3.79 and 4.07 ppm. Previously, we had noted that the *O*-methylation of **23a** under the same conditions resulted instead in the inversion of one of the benzofuran rings, affording the non-symmetrical *partial-cone* **23b** (Figure 6). Thus, the size of the linking methylene chains can have significant effects on the resulting conformers, which are formed [41]. The *O*-benzylation of **28a**, produced the *cone*-benzyloxy[3.1.1]MCP **28c** with no isomerization being observed. This is evident from the ^1^H signals for the bridge methylene hydrogen atoms that are split and appear as a pair of doublets at δ = 3.77 and 4.45 ppm [41]. In principle, these molecules could adopt three possible conformations, which are schematically represented in Figure 12.

Gas-phase DFT computational analysis of the three basic types of conformers of compounds **27a**–**30** was undertaken using the geometry-optimized structures of each of these conformers [41]. The calculated optimized-energy differences (kJ mol^–1^) for **27a**–**30** showed that, of the various conformational isomers of **27a**–**30**, the *cone*-shaped structures are the most favored energetically, in the following order: *cone* > *2-partial-cone* > *1,3-alternate.* For example, the *cone* conformer of **28a** is −13.4 and −23.9 kJ mol^−1^ more stable than its corresponding *2-partial-cone* and *1,3-alternate* conformers, respectively. The findings were also consistent with the experimental evidence that the phenolic hydrogen forms weak intramolecular hydrogen bonding with an oxygen atom of one of the benzofuran rings. The distances between the hydroxyl hydrogen and the benzofuran oxygens are, respectively, 2.014 Å and 3.374 Å, 2.698 Å and 4.253 Å, and 2.010 Å and 3.493 Å for the *cone*, *2-partial-cone* and *1,3-alternate* conformers of **28a**. For the corresponding conformers of **29**, the respective distances are 1.836 Å vs. 3.277 Å, 2.605 Å vs. 3.065 Å, and 3.660 Å vs. 3.836 Å.

## 5. Homooxacalixarene-Analogue MCPs Containing Three Aromatic Rings: “Oxacalix[3]arene” Analogues

**Figure 13:**
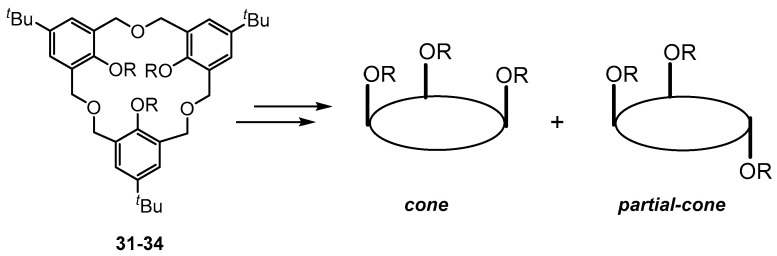
Structures of hexahomotrioxacalix[3]arene **31** (R = H) and schematic representations of the *cone* and *partial-cone* conformers of its tri-*O*-functionalized derivatives **32** (R = alkyl); **33** (R= ethoxycarbonylmethoxy) and **34** (R = *N,N*-diethyl *O*-methylacetamido).

Hexahomotrioxacalix[3]arene **31** (Figure 13; R = H), commonly also called “oxacalix[3]arene”, was first reported in 1962 by Hultzsch who isolated it in less than 1% yield from the reaction of formaldehyde with *p*-*tert*-butylphenol [50], and the evolution of the chemistry of this and related analogues have been well reviewed recently [17,18]. Hexahomotrioxacalix[3]arene **31** can be considered to be an example of a [3.3.3]MCP in which the phenolic units are linked by −CH_2_OCH_2_− bridges. In 1983, Dhawan and Gutsche described detailed reproducible syntheses of **31** [51], which can be conducted in relatively large-scale. Hampton and co-workers also have described an alternative smaller-scale acid-catalyzed procedure which has been used for the synthesis of several different “wide-rim” *para*-substituted analogues of **31** [52]. Shinkai’s group and others have extensively studied **31** as a platform to generate several versatile hosts [53,54,55,56] since, in comparison with the basic calix[4]arene structure, **31** offers some potential advantages: (1) its intra-annular cavity consists of a 18-membered ring, whereas that of calix[4]arene is a 16-membered ring; (2) the rate of ring inversion for derivatives of **31** should be much faster than that for calix[4]arenes because of the flexibility of the dimethyleneoxa linkages; (3) conformational isomerism is much more simplified, as there are only two types of formal conformations possible, namely *cone* and *partial-cone* (Figure 13), in contrast to the four possible formal conformations in calix[4]arenes; (4) the ethereal ring oxygens may act cooperatively with the phenolic oxygens upon binding with metal ions; and (5) its *cone* and *partial-cone* conformers can have C_3v_ and/or C_s_ symmetry, which is particularly useful for the design of receptors for biologically relevant RNH_3_^+^ammonium ions.

In 1993, Shinkai and coworkers reported that treatment of **31** with four alkyl halides in DMF in the presence of NaH, K_2_CO_3_, Cs_2_CO_3,_ *t*-BuOK or K afforded the corresponding *O*-methoxy, *O*-ethoxy, *O*-*n*-propyloxy and *O*-*n*-butyloxy derivatives that can adopt *cone* and/or *partial-cone* conformations (Figure 13) in varying yields ratios. They showed that a metal templating and solvent effect could lead to preferential thermodynamic and/or a kinetic preference for the *partial-cone* conformers of the corresponding tri-*O*-alkylated derivatives. In particular, tri-*O*-*n*-butylated **32** (R – *n*-Bu) was formed exclusively with the use of NaH in DMF but also in a 99:1% ratio over the *cone* conformer when Cs_2_CO_3_ in DMF was used [53]. Shinkai [54,55,56] also reported the synthesis of the *tris*-*O*-ethoxycarbonylmethoxy derivative **33** (R = CH_2_CO_2_Et) by the reaction of excess ethyl bromoacetate with **31** in acetone under reflux conditions with K_2_CO_3_ or Cs_2_CO_3,_ and that an alkali-metal template effect led exclusively to the formation of the *partial-cone*-**33** conformer. With NaH or *t*-BuOK in THF, however, a mixture of *cone* and *partial-cone* products was formed, with the *cone*-**33** conformer being formed in only a 20–22% yield. Shinkai was also able to show both a metal templating and solvent effect for the efficient formation of the tris-*O*-amido derivatives **34** (R = CH_2_CONEt_2_) from the reactions of **31** with diethylchloroacetamide [55]. With NaH in THF, *cone*-**34** was formed exclusively, but, with K_2_CO_3_ or Cs_2_CO_3_ in acetone, *partial-cone-***34** was exclusively formed. With NaH in DMF, a lower overall yield (29 and 26%, respectively) of the *cone* and *partial-cone-***34** was obtained. However, with *t*-BuOK in THF a mixture of *cone* and *partial-cone-***34** products was formed in **60** and 38% overall yield.

**Figure 14:**
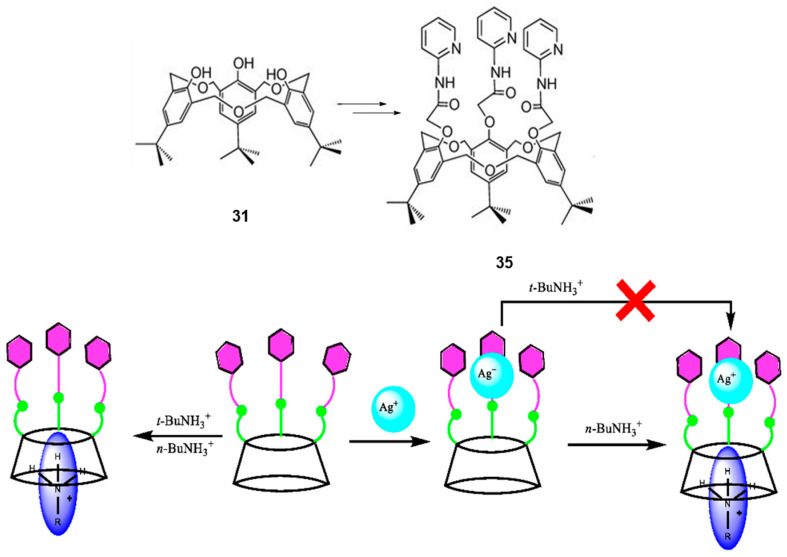
A schematic representation showing the cooperative binding mode of **35** (shown in a cone conformation) with and without Ag^+^ complexation and selective binding with *n*-BuNH_3_^+^ and *t*-BuNH_3_^+^ cations. The green circles represent the CONH− and the hexagons represent the pyridyl groups. (Figure take from [57]).

**Figure 15:**
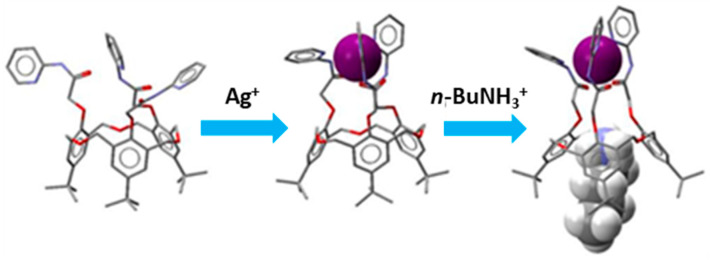
Geometry-optimized (PBE0/LANL2DZ) structures of *cone*-**35** and its complex with Ag+ and *n*BuNH_3_^+^. Left: The free *cone*-**35**, Middle: 1:1 *cone*-**35** ⊃Ag^+^ complex, and right: *n*BuNH_3_^+^ ⊂ [*cone*-35 ⊃Ag^+^] complex. Figure taken from reference [57].

Recently, **35** the *C*_3_-symmetric *N*-pyridyl *O*-methylacetamido functionalized derivative of **31** (Figure 14) was reported to be able to selectively and cooperatively bind Ag^+^ and *n*-butylammonium ions and be controlled by the metal ion [57]. The geometries of the molecular structures were optimized in the gas phase using the PBE0 functional theory with the LANL2DZ basis set. The calculated binding or interaction energies (IE) for *cone-***35** ⊃*n*-BuNH_3_^+^, *cone-***35** ⊃*t*-BuNH_3_^+^, *cone-***35** ⊃Ag^+^, and *n*BuNH_3_^+^ ⊂ [*cone*-**35** ⊃Ag^+^] are −298.8 kJ mol^−1^, −268.3 kJ mol^−1^, −457.1 kJ mol^−1^, and –525.8 kJ·mol^−1^, respectively, and were in agreement with the trend for the complexation data determined by the ^1^H-NMR spectroscopic titration experiments with the corresponding perchlorate salts [57]. A conceptualization of the complexation of *n*BuNH_3_^+^ by the receptor *cone-***35** is shown in Figure 15.

**Figure 16:**
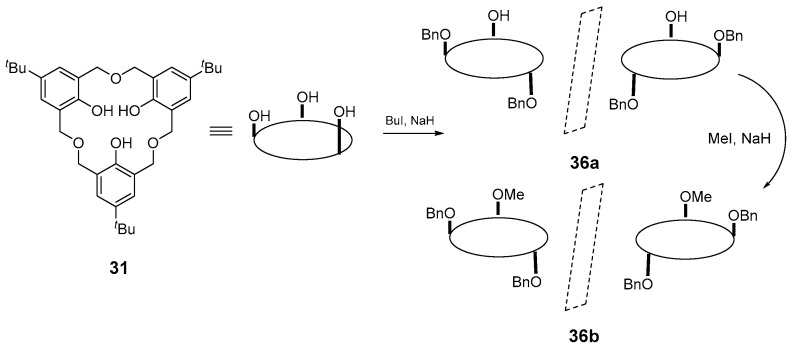
Synthesis of chiral oxacalixarene MCP analogues **36a**–**b** showing the chiral atropisomers. Figure adapted from reference [58].

Chirality is also possible with unsymmetrically substituted hexahomooxacalix[3]arenes. Araki et al. had previously reported the “*pseudo C*_2_-symmetric inherent chirality” of the di-*O*-benzylated derivative **36a** and the di-*O*-benzylated-mono-*O*-methoxy derivative (Figure 16) **36b** of oxacalix[3]arene **31**, by *O*-alkylation at the lower rim of **31** and that these molecules were useful for the recognition of α-amino acid derivatives [58].

In a very recent contribution, Marcos and co-workers reported their latest results with cation and anion ditopic receptors based upon trisubstituted derivatives of **31** with lower-rim *O*-*tert*-butylurea, phenylthiourea or phenylurea functionalities, which are connected to the hexahomotrioxacalix[3]arene scaffold via butyl spacers. [59] Both *cone* and *partial-cone* conformers were obtained with the phenylurea but only *partial-cone* products, as determined by solution NMR studies and their binding properties towards several relevant anions with different geometries were assessed by proton NMR titrations. The *cone* conformation of the *tris*phenylurea derivative was also confirmed by single-crystal X-ray crystallography and also proved to be the best an anion receptor, with the highest affinity being shown toward the trigonal planar acetate and benzoate anions (log *K*_assoc_ = 4.12 and 4.00, respectively) amongst the spherical, linear and tetrahedral anions tested. It also proved to be an effective ditopic receptor for several biogenic amine hydrochlorides monoamine neurotransmitters and trace amine hydrochlorides, in different solvents.

**Figure 17:**
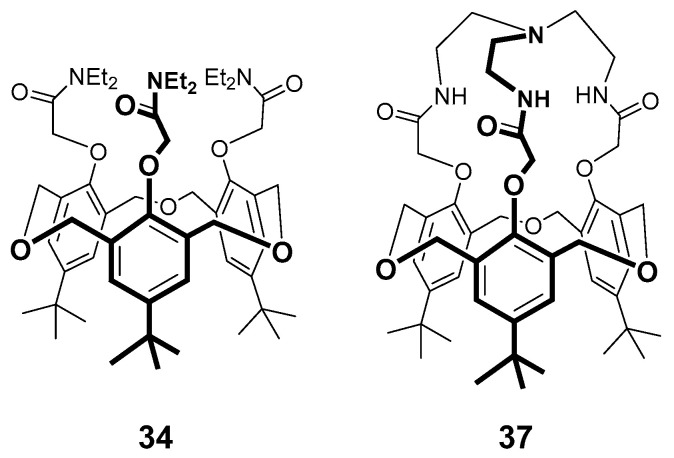
Structures of hexahomotrioxacalix[3]arene-analogue-MCPs **34** and **37**.

Jabin’s group [60] compared the binding of a series of ammonium ions with two previously reported tris-*O*-amido derivatives, **34** [54] and the *tris*-mediated cryptand **37** previously reported by Yamato and coworkers [61] in 10% yields, and later in an improved 50% yield by Jabin and coworkers [62]. Both of these receptors are locked in *cone*-conformations (Figure 17) and ^1^H-NMR studies with both formed endo-complexes with seventeen different primary ammonium ion guests, including biomolecules, even in protic media.

The ammonium ions of each of these guests inserted deeply into the polyaromatic cavity of the host receptor molecules. The NH_3_^+^ moieties were situated very close to the 18-atom lower-rim macrocyclic plane, forming H-bonds with the three oxygen atoms of the macrocycle. The overall topological complementarity between primary ammonium ions and the two cavity-based receptors resulted in the previously unreported complexation specificity for the primary ammonium ions over the secondary, tertiary and quaternary ones that were tested in this study. The authors were able to take advantage of this and demonstrated selective liquid–liquid extraction of primary ammonium salts from water solutions and for the selective recognition of lysine-containing peptides, with obvious potential for peptide sensing. Interestingly, also, **34** showed better binding than **37** presumably since the capping reduced the flexibility for the polyaromatic cavity to accommodate the guests.

**Figure 18:**
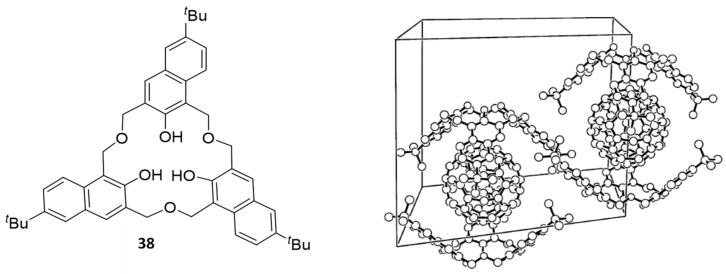
Hexahomotrioxacalix[3]naphthalene **38** and the single-crystal X-ray structure of its 2:1 complex with fullerene-C_60_. Figure adapted from Reference [64].

To date, there is only one report of a 2-naphthol ring-analogue of **31**, namely oxacalix[3]naphthalene **38**, which has its naphthol units linked by -CH_2_OCH_2_- bridges and can be considered to be an example of a [3.3.3]metanaphthalenophane analogue of the more common [3.3.3]homooxacalixarenes described above. This compound that was reported in 2001 by our group, was formed in 25% yield along with its unsymmetrical regioisomer in lower yield, via a fragment-based approach in which the linear precursor underwent cyclo-condensation under acidic conditions [63] similar to those described Hampton [51]. Only a limited study of its complexation properties with metal ions was conducted; however, a ^1^H NMR study of its complexation properties with C_60_ fullerene was reported; additionally, it formed a solid state 2:1 supramolecular complex in its *cone* conformation with C_60_ (Figure 18) [64] in contrast to the 1:1 complexes observed for C_60_ with **31** and several derivatives thereof reported earlier by Tsubaki et al. [65].

## 6. Conclusions

In this review article, we have focused mainly upon the synthesis, structure and conformational properties of calixarene-analogous metacyclophanes, which contain three aromatic rings. These molecules can be considered to be analogues of the relatively less-known “calix[3]arenes”. This group of molecules also encompasses homocalix[3]arenes and homooxacalix[3]arenes and can be considered as homocalix[3]arene- and homooxacalix[3]arene-analogous metacyclophanes. To date, the latter, including their supramolecular properties, have been the most extensively studied. This review highlights the origins, synthesis, structural aspects including their inherent chirality, and some of the different modifications that are possible with these molecules as a group that can lead to diverse application possibilities. We have mostly considered and cited the more recent literature that has been published and, where available, the more recently published reviews. The supramolecular properties of the new synthetic homocalix[3]arene-analogous metacyclophanes, which the Yamato group has mainly been focused on, however, have been untapped and will be subjected to further investigation.

## Figures and Tables

**Table 1:** Influence of substituents on the conformations of benzofurans **25a**–**e** [40].

Compound	*T*_c_	ΔG^≠^ kJ mol^−1^	*Cone:saddle*(−30 °C)
**25a**	40 *^a^*	61.9	80:20
**25b**	45 *^a^*	62.8	100:0
**25c**	28 *^a^*	58.6	40:60
**25d**	50 *^a^*	68.2	100:0
**25e**	75 *^b^*	69.0	20:80

*^a^*: CDCl_3_; *^b^*: CDBr_3_ (300 MHz).
